# Transcriptome of Two-Hybrid Poplar (*Populus alba* × *P. tomentiglandulosa*) During Adventitious Root Formation After Stem Cutting

**DOI:** 10.3390/biology14070751

**Published:** 2025-06-23

**Authors:** Siyeon Byeon, Il Hwan Lee

**Affiliations:** Department of Forest Bioresources, National Institute of Forest Science, Suwon 16631, Republic of Korea; sybyun419@korea.kr

**Keywords:** rooting, transcriptomic analysis, RNA-seq, adventitious roots, propagation

## Abstract

Poplar (*Populus* spp.) is an economically and ecologically important temperate tree species known for its rapid growth. Clonal propagation has facilitated genetic advancements, but it remains challenging due to substantial variations in rooting capacity among poplar species and clones. Poplar clones were divided into two groups based on their rooting ability (high or low), and their transcriptome was analyzed for 3 weeks following stem-cutting propagation to investigate the rooting mechanisms of a hybrid of two fast-growing poplar species (*Populus alba* × *P. tomentiglandulosa*). The root length and area of the high-rooting group were 668.7% and 198.4% greater than those of the low-rooting ability group, respectively (maximum *p* < 0.001). Compared to week 0, genes involved in auxin signaling, cell wall organization, and secondary metabolite biosynthesis were consistently upregulated at 1, 2, and 3 weeks after planting, respectively. The expression of genes associated with cell wall differentiation and flavonoid biosynthesis was greater in the high- than in the low-rooting group at week 2. *MYB* and *AP2/ERF* transcription factors, which regulate flavonoid biosynthesis, as well as chalcone isomerase, a key enzyme in early flavonoid biosynthesis and root formation, were upregulated in the high-rooting group. The flavonoid biosynthesis pathway is important in rooting after stem cutting of *Populus alba* × *P. tomentiglandulosa* hybrids.

## Simple Summary

Poplars are rapidly growing trees and are important both ecologically and industrially. Variations in rooting ability exist among poplar clones of the same species, which significantly impact the success rate of propagation by cuttings. We selected clones of hybrid poplar species that showed high or low root growth and studied their gene activity for 3 weeks after stem cutting. The group with higher rooting capacity exhibited longer and larger roots, as well as elevated expression of genes related to plant hormones, cell wall development, and natural compounds such as flavonoids, compared with the group with lower rooting capacity. Flavonoid production-associated genes may play key roles in root development. These results are expected to improve our understanding of key genes and biological pathways involved in poplar rooting and contribute to the selection of poplar clones with superior rooting efficiency for vegetative propagation by cuttings.

## 1. Introduction

Poplar (*Populus* spp.) is an important economic and ecological tree species in temperate regions worldwide because of its adaptability, rapid growth, substantial woody biomass, and the industrial versatility of its timber [1,2]. Poplar wood is a renewable carbon source in bioenergy and biofuel production. *Populus* has been established as a model genus for forestry research due to its fully sequenced genome, ease of manipulation, short rotation cycle, and ease of vegetative propagation [3,4]. Clonal propagation can accelerate advancements in poplar genetics that facilitate a quick return on investment [5].

Cutting is a vegetative propagation technology that is widely used for poplar breeding and planting [6,7,8,9]. Cuttings from the parent plant can be easily and cost-effectively used to quickly increase individual nursery production [10,11,12]. Adventitious roots form after cutting in response to the injury of various plant organs such as the stems and leaves [7]. The cutting site (i.e., leaf, stem, or root) varies depending on propagation objectives and plant species. Stem cutting is the most common propagation method because of the resulting rapid propagation, reduced cost, and ease of manipulation.

The rooting capacity of the cuttings derived from different *Populus* species, families, and clones varies genetically [9,13]. For instance, root formation substantially differed within 14 days among three *Populus* spp. (*P. alba*, *P. tomentosa* and *P. davidiana*), with *P. alba* exhibiting the highest rooting ability [14]. Root formation was observed 4, 10, and 10 days after stem cutting in *P. nigra*, *P. alba*, and *P. tremula*, respectively. Rooting characteristics also varied among 21 clones of *Populus* species owing to a combination of genetic variations and environmental conditions [15]. Adventitious roots play a critical role in the survival of plants under biotic and abiotic stresses as well as in their adaptation after planting [16,17,18]. For successful propagation after cutting, it is important to select genotypes (clones) with a high propensity for root formation and identify the relevant genes.

Numerous quantitative trait loci (QTLs) are related to root formation, such as root length, diameter, and volume [19,20,21,22,23,24]. In addition, transcriptomic studies have identified candidate genes involved in *Populus* root formation [7]. The differentially expressed genes (DEGs) located within root formation-associated regions, derived from either QTLs or genome-wide associations, can be identified by analyzing the transcriptomes of individuals exhibiting superior and inferior rooting ability [21]. Multiple identified QTLs confirm that adventitious rooting in *Populus* spp. is a quantitative trait governed by specific regions and that plants with different genetic backgrounds should differ in rooting. In addition, gene expression levels widely vary with the time after cutting. Therefore, gene expression patterns should be examined over a time course to gain a better understanding of the root development process during poplar cutting propagation.

*P. alba* has been extensively used in short-rotation plantations for producing timber and pulp. *P. tomentiglandulosa* is a hybrid of *P. alba* and *P. davidiana*, and is widely distributed in South Korea. *P. tomentiglandulosa* can be planted in low-altitude regions such as valleys and mountain foothills. We developed a poplar hybrid with superior growth that is suitable for mountain afforestation by crossbreeding *P. alba* and *P. tomentiglandulosa*. The rooting ability of the F_1_ hybrids of *P. alba* and *P. tomentiglandulosa* varied. As such, candidate genes involved in root formation at a specific phase after cutting needed to be identified for the successful propagation of these poplar hybrids. We divided the hybrids of two fast-growing poplar species (*P. alba* × *P. tomentiglandulosa*) into two groups based on their rooting ability, high and low, after stem cutting. We then comparatively analyzed for three weeks the transcriptome of the groups to identify the genes and pathways crucial for root formation after stem cutting.

## 2. Materials and Methods

### 2.1. Experimental Site and Plant Materials

Six poplar clone (*Populus alba* × *P. tomentiglandulosa*) hybrids (F1) were propagated via stem cutting in pots containing a soil and sand mixture with 40 individuals per clone in April 2020. There were no apparent differences in leaf or branch morphology between the high- and low-rooting groups based on visual observation (Figure S1). The plants were grown in a greenhouse located at the National Institute of Forest Science in Suwon, Korea (37°15′04″ N, 136°57′59″ E). Plants were cultivated in a greenhouse under natural light, with the temperature maintained at 18–25 °C. Their rooting ability was studied for four weeks after planting, and the hybrid clones were divided into high- and low-rooting groups based on the measured rooting length and area. Root length and area were measured four weeks after planting using ImageJ v1.8.0. *T*-tests were conducted to assess the differences between the groups. These clones were replanted via stem cutting under the conditions described above in this section. Samples were collected from the three hybrid clones in each group from the belowground cambium layers 0, 1, 2, and 3 weeks after planting. The samples were preserved in a deep freezer set at −80 °C until RNA extraction.

### 2.2. RNA Extraction and cDNA Library Construction

For high-quality, intact total RNA extraction, tissues excluding the heartwood were harvested from the belowground part of the cuttings after making a shallow, longitudinal incision with a sterile scalpel. RNA was isolated from the cambium layers using a Ribospin Plant Kit (GeneAll, Seoul, Republic of Korea). The integrity of the extracted RNA was evaluated using Bioanalyzer RNA Pico 6000 Chip Library Preparation (Agilent Technologies, Santa Clara, CA, USA) for RNA sequencing. Only RNA samples with RNA integrity numbers (RINs) exceeding 7 were used for cDNA library preparation. A TruSeq Standard mRNA LT Sample Prep Kit was used to assemble paired-end cDNA libraries. These libraries were sequenced on an Illumina NovaSeq 6000 platform (Illumina, San Diego, CA, USA), producing paired-end reads of 101 bp.

### 2.3. RNA-Seq Read Processing

First, the raw data were trimmed using Trimmomatic v0.39 with standard settings to eliminate inferior-quality reads [25]. Second, de novo assembly was conducted using Trinity assembler v2.15.1, employing the default settings to construct a reference transcriptome. Third, the reads filtered from each sample were aligned to the established reference transcriptome using Salmon software version 1.8 [26]. Finally, the DEGs were identified using DESeq2 1.34.0, applying a threshold false discovery rate (FDR) of <0.05 and a log_2_ fold change (log_2_FC) of ≥1 [27].

### 2.4. Gene Ontology Enrichment Analysis

The sequences were aligned with those from the *Arabidopsis thaliana* protein database using BLASTX, a basic local alignment search tool for proteins (https://blast.ncbi.nlm.nih.gov/Blast.cgi, accessed on 11 September 2024), using an e-value cutoff of 10^−7^ for functional annotation identification [28] (Supplementary Data S1). Gene Ontology (GO) terms were analyzed using the PANTHER GO biological process classification system by applying Fisher’s exact test with an FDR threshold of less than 0.05 (http://go.pantherdb.org/webservices/go/overrep.jsp, accessed on 11 September 2024). The pathways were mapped using the Kyoto Encyclopedia of Genes and Genomes (KEGG) database [29].

### 2.5. Validating RNA-Seq Results Using qRT-PCR

Total RNA was isolated using a Ribospin Plant Kit (GeneAll, Seoul, Republic of Korea), and cDNA was synthesized using EcoDry Premix (Oligo dT) kit (Takara Bio, Kusatsu, Japan), according to the manufacturer’s instructions. The RT-qPCR primers were designed using Primer 3 (https://www.primer3plus.com/, accessed on 23 September 2024). Seven genes were selected to validate the RNA-seq results, with ubiquitin (UBQ) serving as the normalization reference gene (Table S1) [30]. The qRT-PCR was analyzed on a CFX96 Touch Real-Time PCR Detection System (Bio-Rad Laboratories, Hercules, CA, USA), with thermal cycling conditions set at 95 °C for 10 min, followed by 40 cycles of 95 °C for 15 s, and 60 °C for 1 min. Each reaction mix consisted of 10 µL of IQ Sybr Green SuperMix (Bio-Rad Laboratories), 1 µL of cDNA, 1 µL of 10 nM gene-specific primers (both forward and reverse), and 7 µL of DNase- and RNase-free water with a total volume of 20 µL. Three biological replicates were used for each treatment and cloning. The 2^−ΔΔCt^ method was used for quantifying the gene expression ratios [31]. Pearson correlation analysis was performed to validate consistency between RNA-seq and qRT-PCR measurements.

### 2.6. Data

The RNA-seq data were deposited in the NCBI SRA database (BioProject accession number PRJNA1106192).

## 3. Results

### 3.1. Phenotype of High- and Low-Rooting Ability Poplar Groups

**Figure 1:**
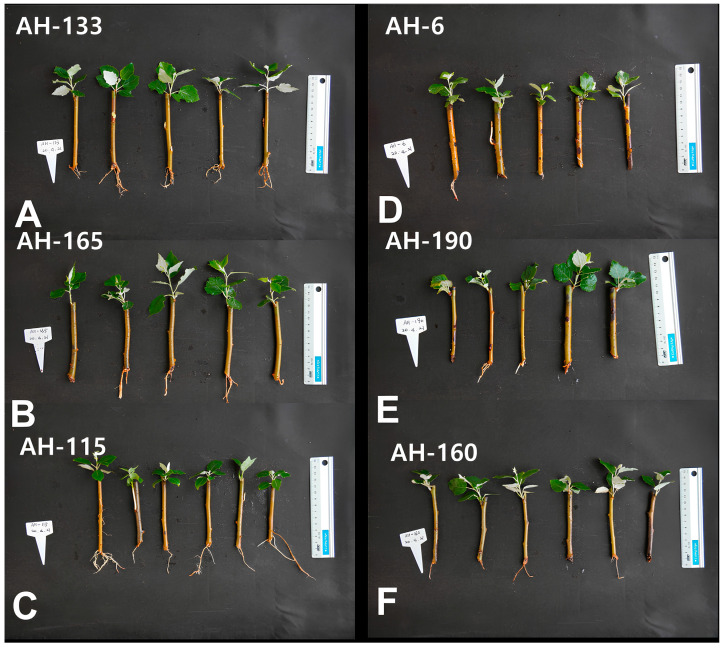
The high-rooting ability group ((**A**): AH-133, (**B**): AH165, (**C**): AH 115) and low-rooting ability group ((**D**): AH-6, (**E**): AH-190, (**F**): AH-160) 4 weeks after plantation.

**Figure 2:**
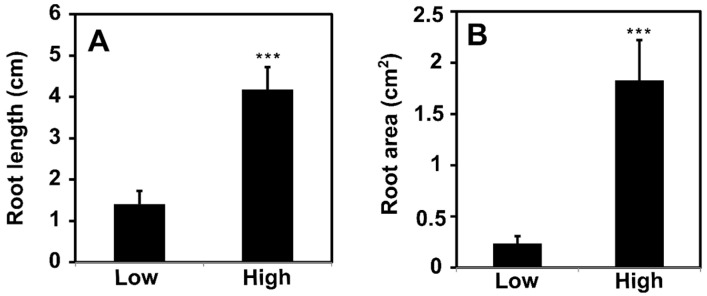
(**A**) The root length (High: 1.83 ± 0.38 cm^2^ vs. Low 0.23 ± 0.07 cm^2^) and (**B**) root area (High: 4.18 ± 0.52 cm vs. Low 1.40 ± 0.52 cm) of high- and low-rooting ability groups 4 weeks after plantation. Bar indicates mean of root length and root area in each group. Error bars represent standard error. Statistical significance at *p* < 0.001 is indicated by *** (Welch’s *t*-test).

The rooting ability of the AH-133, AH-165, and AH-115 clones was stronger than that of AH-6, AH-190, and AH-160 at 4 weeks after planting (Figure 1). The roots in the high-rooting ability group were longer (+668.7%, *p* < 0.001) and had a larger area (+198.4%, *p* < 0.001) than those in low-rooting ability group (Figure 2A,B).

### 3.2. De Novo Assembly of High- and Low-Rooting Ability Poplar Groups

We comparatively analyzed the transcriptome between the high- and low-rooting groups at different time points after cutting to identify the genes related to root formation in poplar hybrids. Samples of the belowground cambium layers were collected 0, 1, 2, and 3 weeks after cutting from three clones of the high-rooting group and the low-rooting group. The RNA was sequenced using these samples. A total of 851,225,594 raw reads from 128,535,064,694 bp were generated. We filtered the raw reads, generating 589,964 transcripts and 379,983 genes from 204,796,926 reads that were assembled using Trinity. The average transcript contig length and N50 were 538.96 and 1362 bp, respectively (Table 1).

### 3.3. Identifying DEGs Between High- and Low-Rooting Poplar Groups

**Figure 3:**
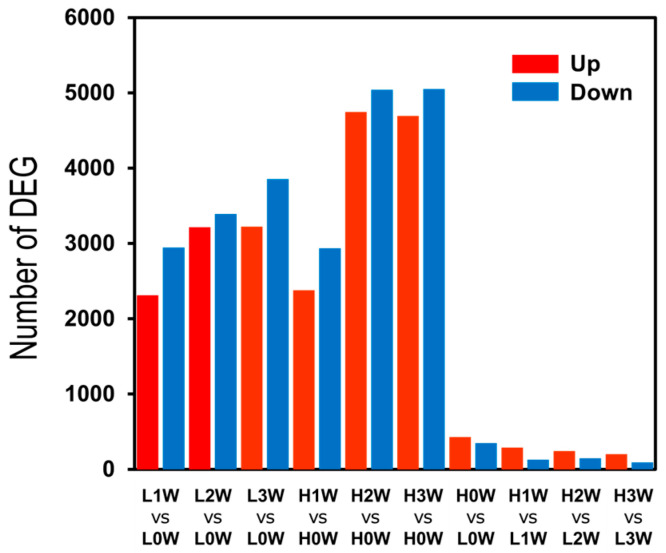
The number of differentially expressed genes in each comparison. H and L represent high and low group in rooting, respectively. The 0 W, 1 W, 2 W, and 3 W indicate weeks after plantation. Red and blue colors represent up- and downregulated DEGs, respectively. DEGs were identified using a threshold of false discovery rate (FDR) < 0.05 and |log_2_ FC| ≥ 1.

A total of 2312, 3214, and 3223 DEGs were upregulated and 2946, 3391, and 3856 DEGs were downregulated in the low-rooting group (L) at 1, 2, and 3 weeks after cutting compared with 0 weeks, respectively (L1W vs. L0W, L2W vs. L0W, and L3W vs. L0W) (Figure 3, Supplementary Data S2). A total of 2379, 4746, and 4695 upregulated DEGs and 2935, 5040, and 5052 downregulated DEGs were found in the high-rooting group (H) in H1W vs. H0W, H2W vs. H0W, and H3W vs. H0W, respectively. A total of 430, 289, 242, and 201 DEGs were upregulated in the H0W vs. L0W, H1W vs. L1W, H2W vs. L2W, and H3W vs. L3W comparisons, respectively. A total of 350, 129, 146, and 92 DEGs were downregulated in the H0W vs. L0W, H1W vs. L1W, H2W vs. L2W, and H3W vs. L3W groups, respectively.

### 3.4. GO Enrichment Analysis of DEGs Between High- and Low-Rooting Poplar Groups

We conducted GO enrichment analysis to identify the biological processes in which the DEGs were enriched between the high- and low-rooting groups (Supplementary Data S3). The upregulated DEGs in L1W vs. L0W, L2W vs. L0W, and L3W vs. L0W were enriched in GO terms related to root morphogenesis, root system development, root development, secondary metabolite biosynthetic processes, phenylpropanoids, lignin biosynthetic processes, cell wall organization, and cell division (Table S2). The downregulated DEGs in the same comparison were enriched in the photosynthesis, chloroplast, carbon fixation, and light-reaction-related GO terms. The downregulated DEGs were enriched in photosynthesis-related GO terms in the H1W vs. H0W, H2W vs. H0W, and H3W vs. H0W groups (Table S3). The upregulated DEGs in the same comparison were enriched in the root development, cell division, and secondary metabolite biosynthesis, such as lignin and phenylpropanoid, GO terms. The genes related to auxin transport were upregulated in the first week, whereas those associated with fluid transport, water transport, vesicle-mediated transport, and phloem transport were upregulated in the second week in the low-rooting group. In contrast, the GO terms related to water and fluid transport, xylem development, and xylem and phloem pattern formation were enriched in the first week in the high-rooting group.

**Figure 4:**
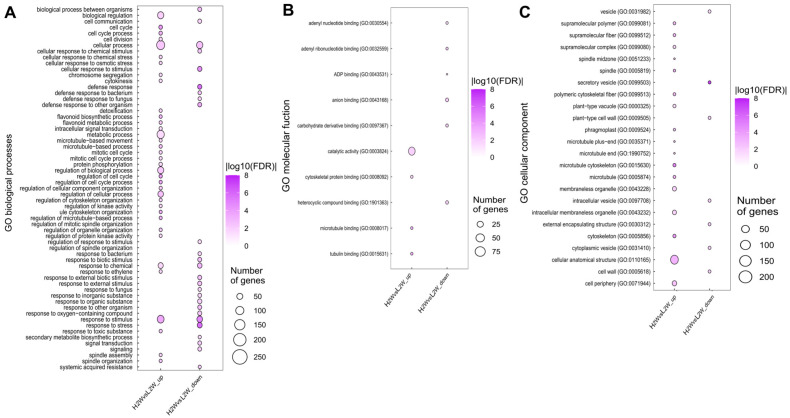
Gene Ontology (GO) enrichment analysis of up- and downregulated differentially expressed genes (DEGs) in the high-rooting group compared to the low-rooting group two weeks after plantation (H2W vs. L2W). GO terms from three categories—(**A**) biological process (BP), (**B**) molecular function (MF), and (**C**) cellular component (CC)—are shown. Genes were selected for enrichment analysis based on FDR < 0.05 and |log_2_FC| ≥ 1. Bubble color represents the −log_10_ FDR value, and bubble size indicates the number of DEGs associated with each GO term.

**Figure 5:**
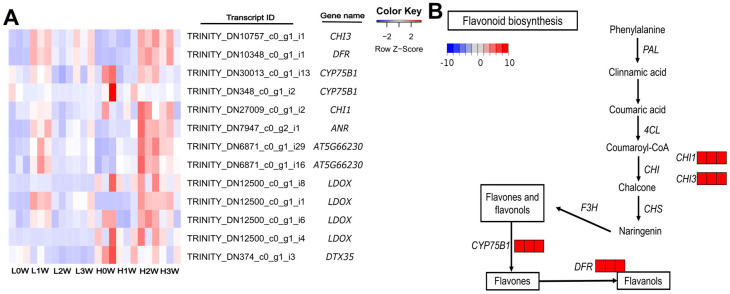
(**A**) Heatmap of upregulated genes related to flavonoid biosynthesis in high group in rooting 2 weeks after plantation. Heatmap colors indicate the Z-scores of TMM−normalized TPM values. The red and blue colors indicate a higher and lower expression of the gene, respectively. (**B**) Flavonoid biosynthesis pathway in the high-rooting group compared to the low-rooting group at 2 weeks after stem cutting. Upregulated genes (log_2_FC ≥ 1) are shown in red, and downregulated genes (log_2_ FC ≤ −1) in blue.

Enrichment analysis of upregulated DEGs in the H2W vs. L2W comparison identified 38 GO biological process (BP) terms, including cell division, cell cycle, flavonoid biosynthetic and metabolic processes, mitotic cell cycle, spindle assembly, and spindle organization (Figure 4A). Furthermore, 17 cellular components (CC) terms, including microtubule and spindle, and 4 molecular function (MF) terms, such as microtubule binding, were enriched among these genes (Figure 4B,C, Supplementary Data S4). In contrast, GOBP terms related to defense and various other responses were enriched in the downregulated DEGs in the same comparison. Additionally, GOCC terms such as vesicle and cell wall, as well as GOMF terms including adenyl nucleotide binding and anion binding, were also enriched in these downregulated DEGs. One GO term was identified for the upregulated DEGs in H3W vs. L3W, and no GO term was substantially enriched with the downregulated DEGs. Fatty acid degradation and flavonoid biosynthesis were identified in H2W and L2W, respectively, in the KEGG analysis (Table 2). The genes involved in flavonoid biosynthesis included chalcone isomerase (*CHI*), cytochrome P450 75B1 (*CYP75B1*), dihydroflavonol-4-reductase (*DFR*), leucoanthocyanidin dioxygenase (*LDOX*), and anthocyanin reductase (*ANR*) (Figure 5A). Notably, *CHI*, *CYP75B1*, and *DFR* were significantly upregulated in the high-rooting group compared to the low-rooting group 2 weeks after stem cutting (Figure 5B).

### 3.5. Rooting Ability-Related Transcription Factors

**Figure 6:**
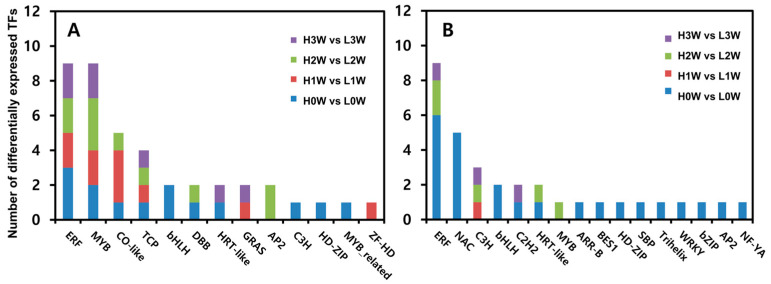
The number of differentially expressed (**A**) upregulated and (**B**) downregulated transcription factors (TFs) in each comparison. H and L represent high and low group in rooting, respectively. 0 W, 1 W, 2 W, and 3 W indicate weeks after plantation. Differentially expressed TFs were identified using a threshold of false discovery rate (FDR) < 0.05 and |log_2_ FC| ≥ 1.

We identified the transcription factors (TFs) that could be involved in regulating root formation in these hybrid poplars. The largest groups of TFs among the upregulated genes were *ERF* (nine genes) and *MYB* (nine genes), followed by *CO-like* (five genes), *TCP* (four genes), and other TF families (Figure 6A, Supplementary Data S5). The *MYB (MYB82*, *MYB62*, and *MYB6*), *ERF* (*ERF9* and *ERF59*), and AP2 (*PLT2* and *BBM*) families were upregulated in the high-rooting group compared with the low-rooting group 2 weeks after planting. *ERF* (nine genes) was the largest group of the downregulated TFs (Figure 6B), among which six *ERF* genes were downregulated in the high-rooting group compared with the low-rooting group 0 weeks after planting. *ERF* (two genes) was the largest group of the downregulated DEGs, followed by *MYB*, *C3H*, and *HRT-like* (one gene) 2 weeks after planting in the high-rooting compared with the low-rooting group.

### 3.6. qRT-PCR Validation of DEG Expression Levels

**Figure 7:**
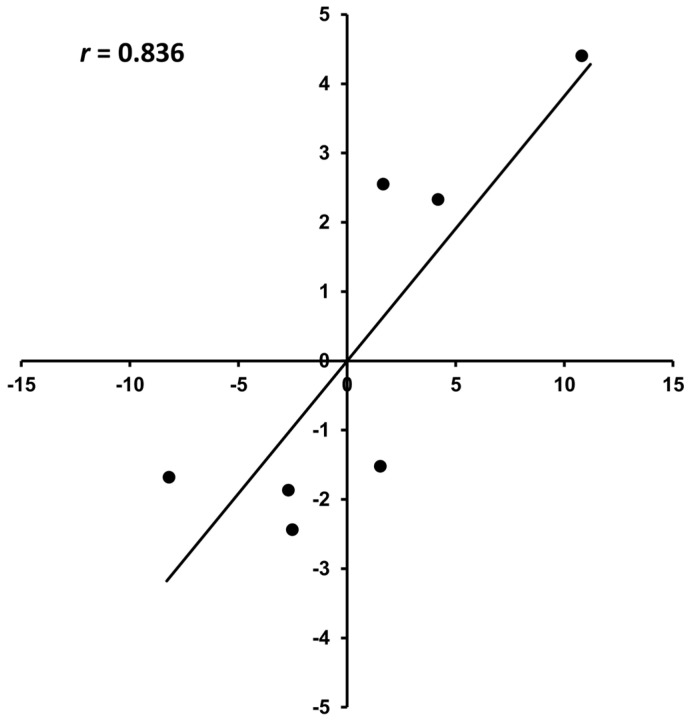
The qRT−PCR validation of differentially expressed genes in hybrid poplar (*Populus alba* × *P. tomentiglandulosa*) between the high-rooting ability group (H) and low-rooting ability group (L) at 2 weeks after plantation. The correlation between the log_2_FC analyzed by RNA-Seq (*x*-axis) and the data obtained from real-time PCR (*y*-axis) was assessed for both the high- and low-rooting ability groups. Pearson correlation analysis showed a strong positive correlation between RNA-seq and qRT-PCR data (r = 0.836, *p* = 0.019), supporting the consistency of expression patterns. FPKM values represent normalized transcript abundance from RNA-seq. qRT-PCR log_2_ FC were calculated using the −ΔΔCt method.

We performed qRT-PCR on the seven selected genes to validate the RNA-seq results (Figure 7). The log_2_FC between the RNA-seq data and qRT-PCR results was significantly correlated (*p* = 0.019), specifically when comparing the H2W and L2W conditions (r = 0.836).

## 4. Discussion

The genetic variability affecting the rooting ability of cuttings from various *Populus* families, species, and clones has been extensively studied for over half a century [32]. We also observed considerable differences in root length and area among the *Populus alba* × *P. tomentiglandulosa* F_1_ hybrid clones (Figure 1 and Figure 2). The roots of the high-rooting ability group were significantly longer by 668.7% and larger by 198.4% than those of the low-rooting ability group (Figure 2).

We comparatively analyzed the transcriptome of the high- and low-rooting groups of the F_1_ progeny of hybrid poplar clones to identify the genes and biological processes related to root formation and development after stem cutting. The development of adventitious roots from stem cuttings is categorized into multiple stages based on physiological and metabolic indicators: first, dedifferentiation, where cells respond to the root-inducing signal (auxin); second, cell division; third, the emergence of root primordia from the stem. Auxins play a pivotal role in the formation of adventitious roots from the stem cuttings of various plant species [33,34,35]. The expression levels of genes related to auxin, cell wall organization, and secondary metabolite biosynthesis processes, such as phenylpropanoids, lignin, and flavonoids, were higher in weeks 1, 2, and 3 than in week 0 in both groups, whereas the expression levels of photosynthesis-related genes decreased over time (Tables S2 and S3). However, the expression of genes related to xylem development increased more rapidly in the high-rooting group than in the low-rooting group. Extensive cell division occurs during adventitious root formation within the vascular tissues of the petiole, including the xylem, leading to the formation of a microcallus, which is crucial for specifying founder cells [36]. In particular, GO terms related to the mitotic spindle, microtubule (CC), and microtubule binding (MF) were enriched in the upregulated genes in the high-rooting group compared with the low-rooting group 2 weeks after cutting. It suggested enhanced cytoskeletal activity associated with cell division during early stages of adventitious root development (Figure 4B,C). This observation is consistent with previous studies showing that spindle and microtubule dynamics play critical roles in root development.

EB1 family members, specifically EB1b and EB1c, are known to be associated with spindle and microtubules. These microtubule-end-binding 1 (EB1) proteins play a critical role in directional root growth in Arabidopsis thaliana by promoting the organized arrangement of cortical microtubules in elongating epidermal cells [37,38]. In this study, homologs of these genes were also found to be upregulated in the high-rooting group compared to the low-rooting group 2 weeks after cutting. This finding suggests that the upregulated expression of these genes might positively regulate adventitious root development by promoting microtubule organization and cell division in the high-rooting group. The expression levels of genes related to secondary metabolites, including phenylpropanoid and flavonoid biosynthesis, increased after cutting, facilitating cell wall organization for adventitious root initiation, whereas the expression levels of photosynthesis-related genes decreased in woody plants such as *Pinus contorta*, *Vigna radiata*, and *Cryptomeria japonica* [39,40,41].

The phenylpropanoid pathway is a pivotal metabolic network in plants, being responsible for biosynthesizing a diverse array of secondary metabolites, including lignins and flavonoids [42,43]. Lignin, a complex phenolic polymer synthesized through the phenylpropanoid pathway, is a primary component of the cell walls in tracheary elements [44,45]. Additionally, lignin enhances root development by strengthening the cell walls during the sealing process, thereby increasing the strength and stability of the roots, which are essential for plant survival and growth [46]. Flavonoids are phenolic compounds that function as signaling molecules and are involved in root formation by regulating auxin transport [47,48]. Flavonoids concentrate in the dividing cells located in the root [49,50] and play a crucial role in adventitious root formation during cutting propagation in various tree species [51,52].

In this study, flavonoid biosynthesis played a crucial role in the root formation of F_1_ *Populus alba* × *P. tomentiglandulosa* hybrids. The expression levels of flavonoid-biosynthesis- and cell division-related genes were higher in the high-rooting than in the low-rooting group 2 weeks after planting (Figure 4A). In addition, the results of KEGG pathway enrichment analysis showed that flavonoid biosynthesis was the only considerably upregulated pathway in the high-rooting group compared with the low-rooting group 2 weeks after planting (Table 2). The expression levels of key genes in the flavonoid biosynthesis pathway, such as *CHI*, *CYP75B1*, *DFR*, *LDOX*, and *ANR*, were upregulated in the high-rooting group 2 weeks after planting (Figure 5).

TFs play important roles in regulating gene expression during various aspects of plant development. The transcription factors (TFs) *MYB*, *ERF*, and *AP2* were upregulated in the high-rooting group 2 weeks after cutting (Figure 6). These TFs are well known for their regulatory roles in flavonoid biosynthesis in various plant species such as *Arabidopsis thaliana*, *Onobrychis viciifolia*, *Erigeron breviscapus*, and *Camellia sinensis* (L.) [53,54]. In particular, the *AP2/ERF* family has been shown to activate the flavonoid pathway through modulation of *CHI* gene expression in red pear, citrus, and other species [55,56]. *CHI* catalyzes an early step of the flavonoid biosynthesis pathway, with the highest gene expression levels occurring in the root tips and young roots [57,58,59]. The silencing of *CHI* flavonoid biosynthesis genes reduced root nodulation in *Medicago truncatula* [19]. The overexpression of *CHI* increased flavonoid production in the hairy roots of *Glycyrrhiza uralensis* and *Scutellaria baicalensis* [59,60]. In contrast, the *CHI* transcripts and flavone production were lower in *CHI*-silenced hairy root lines than in the wild type [59]. The expression of *CHI* genes was upregulated in the high-rooting group compared with the low-rooting group 2 weeks after cutting.

The *AP2* TFs, *PLT2* and *BBM*, were upregulated in the high-rooting group 2 weeks after cutting. These TFs are directly involved in the auxin-responsive root developmental pathway [55,61,62]. *BBM* overexpression induces YUCCA-dependent auxin biosynthesis and promotes the spontaneous development of somatic embryos and cotyledon-like structures in *Arabidopsis* and apples [63,64]. Additionally, *PLT2* is essential for the specification and maintenance of stem cells, and its regulation is influenced by an auxin maximum located distal to vascular precursors [64]. Imin, Nizamidin, Wu, and Rolfe [55] further reported that *WOX5*, *PLT1*, *PLT2*, and *BBM* strongly respond to the presence of auxin and are highly expressed in 1-week-old root-forming calli in *Medicago truncatula*. This suggests that *AP2* TFs such as *PLT2* and *BBM* regulate root development in response to auxins after cutting in this poplar hybrid.

Extensive studies on *Populus* species have highlighted the roles of *AP2/ERF* transcription factors and flavonoid-related pathways in adventitious root formation following cutting [7]. The auxin receptors *TIR1* and *PagFBL1* are regulators of adventitious rooting and interact with *IAA28* in the *P. alba* × *P. glandulosa* hybrid [65]. Consistent with our findings, Rigal et al. [66] reported that *AP2/ERF* family members are crucial for controlling adventitious rooting in *P. trichocarpa*, suggesting their involvement in the rooting process of poplar cuttings. The role of phenolic compounds in the formation of adventitious roots is established: they induce rooting by inhibiting IAA decarboxylation during the initial stages, similar to auxin [67,68,69]. The external application of phenolic compounds such as pyrogallol and salicylic acid enhanced root development in various clones of *P. alba* and *P. canescens* [70].

Together, these results suggest that TFs contribute to enhanced rooting ability through two converging mechanisms: (1) activation of flavonoid biosynthesis, which facilitates auxin transport and ROS buffering, and (2) direct regulation of auxin-responsive developmental pathways via *AP2* TFs such as *PLT2* and *BBM.* This coordinated transcriptional response provides a mechanistic explanation for the observed differences in rooting performance between high- and low-rooting groups.

## 5. Conclusions

We observed differences in the root length and area among *Populus alba* × *P. tomentiglandulosa* F_1_ hybrid clones when propagated via stem cutting. We comparatively analyzed the transcriptome after stem cutting and found that genes related to auxin, cell wall composition, and secondary metabolite processes were upregulated in weeks 1, 2, and 3 after planting compared with week 0. Notably, the expression levels of genes related to cell wall differentiation and flavonoid biosynthesis were markedly higher in the high-rooting group than in the low-rooting group in week 2. The expression levels of transcription factors *MYB* and *AP2/ERF*, which activate flavonoid biosynthesis, as well as that of *CHI*, a key enzyme in the flavonoid pathway, were more highly expressed in the high-rooting group 2 weeks after cutting. These results are consistent with the findings obtained for other poplar species, confirming that the flavonoid biosynthesis pathway plays a crucial role in rooting following stem cutting in *Populus* spp. We identified crucial genes and biological pathways related to root development after poplar cutting propagation, which can be used to guide applications for increasing the efficiency of poplar propagation and improving the development of varieties with high rooting ability.

## Figures and Tables

**Table 1:** Transcriptome summary statistics from de novo assembly.

Assembled Contigs	No.
Total Trinity genes (*n*)	379,983
Total Trinity transcripts (*n*)	589,964
GC content (%)	43.44
Contig N50 length (bp)	1362
Average contig length (bp)	538.96
Total assembled bases	204,796,926

**Table 2:** Kyoto Encyclopedia of Genes and Genomes pathway analysis of upregulated differentially expressed genes in the high-rooting ability group compared to low-rooting ability group at different time points after stem cutting plantation.

	KEGG Pathway	Gene	FDR	Rich Factor (%)
H0W vs. L0W	Fatty acid degradation	7	0.018	1.84
H1W vs. L1W	n.s.			
H2W vs. L2W	Flavonoid biosynthesis	6	<0.001	2.75
H3W vs. L3W	n.s.			

Differentially expressed genes were identified using a false discovery rate (FDR) threshold of <0.05 and absolute log_2_ fold change |log_2_FC| ≥ 1. H and L represent high and low group in rooting, respectively. 0 W, 1 W, 2 W, and 3 W indicate weeks after plantation. n.s. means that it is statistically non-significant.

## Data Availability

The data presented in this study are openly available in [NCBI SRA] at [PRJNA1106192].

## Supplementary Materials

The following supporting information can be downloaded at: https://www.mdpi.com/article/10.3390/biology14070751/s1, Table S1: Primer sequences used to validate RNA-Sequencing results of hybrid Poplar (*Populus alba* × *P. tomentiglandulosa*) between high rooting ability group (H) and low rooting ability group (L) at 2 weeks after plantation.;Table S2: Gene ontology (GO) analysis of up- and down-regulated differentially expressed genes (DEGs) in low rooting group (L) compared to low group 0 week after plantation (L0W).;Table S3: Gene ontology (GO) analysis of up- and down-regulated differentially expressed genes (DEGs) in high rooting group (H) compared to low group 0 week after plantation (H0W).;Table S4: Comparison of gene expression between high- and low-rooting groups based on RNA-seq and qRT-PCR analyses.;Figure S1: Representative images of (A) branch and leaves of high rooting group, (B) leaf of the high rooting group, (C) branch and leaves of the low rooting group, and (D) leaf of the low rooting group.; Supplementary Data S1: Blastx results of de novo assembled transcripts using the *Arabidopsis thaliana* protein database; Supplementary Data S2. The number of differentially expressed genes in each comparison.; Supplementary Data S3: List of Differentially Expressed Genes in the High Rooting Group Compared to the Low Rooting Group at 2 Weeks After Plantation; Supplementary Data S4: The number of differentially expressed genes in each comparison.; Supplementary Data S5: List of differencially expressed Transciption factors.
