# Supplementary material for: Transcriptome of Two-Hybrid Poplar (Populus alba × P. tomentiglandulosa) During Adventitious Root Formation After Stem Cutting
Source: Biology (Basel). 2025 Jun 23;14(7):751. doi: 10.3390/biology14070751 (PMC12293016; doi:10.3390/biology14070751)
Supplement: Supplementary file 1 [file biology-14-00751-s001.zip › Supplementary Table&Figure.pdf]

Table S1. Primer sequences used to validate RNA-Sequencing results of hybrid Poplar (*Populus alba* × *P. tomentiglandulosa*) between high rooting ability group (H) and low rooting ability group (L) at 2 weeks after plantation.

**Gene**	**Forward primer**	**Reverse primer**
TRINITY_DN5565_c0_g1_i3	CAAAATGGCCCAGGACAAGC	CTGCATGAGCGCCTTCATTC
TRINITY_DN10757_c0_g1_i1	GGGTCGTGGTGATCAAGGAG	CGATCTTCTCCAGGGCTTCC
TRINITY_DN16489_c0_g1_i2	GTGTGTGGGAGGATGGATCG	TATCAGCTGCCTTGGCAGAC
TRINITY_DN3440_c0_g1_i15	AAACGCCTTGTTGGAATGGC	CAGCTTCAGCCGTTGTTGAC
TRINITY_DN6189_c0_g1_i11	CAAAAGGGTGTCCTCCTCCC	GGTGTCTTGCTTTGGCTTCG
TRINITY_DN4210_c0_g1_i63	CATCCGGGAACTGCTGTCTT	CGGCAACTGAGTCTGCTACA
TRINITY_DN10348_c0_g1_i1	CAGGTGCTGTGGAAACATGC	GTGCATAGCTTGGGCGATTG
Ubiquitin	GTTGATTTTTGCTGGGAAGC	GATCTTGGCCTTCACGTTGT

Table S2. Gene ontology (GO) analysis of up- and down-regulated differentially expressed genes (DEGs) in low rooting group (L) compared to low group 0 week after plantation (L0W).

	GO biological process complete	Gene	FDR
L1W vs L0W up	anatomical structure development (GO:0048856)	466	1.02E-14
	developmental process (GO:0032502)	484	2.46E-13
	multicellular organism development (GO:0007275)	380	3.31E-11
	system development (GO:0048731)	334	4.06E-11
	plant organ development (GO:0099402)	209	2.97E-10
	tissue development (GO:0009888)	189	6.34E-09
	cell wall organization or biogenesis (GO:0071554)	112	9.08E-09
	secondary metabolite biosynthetic process (GO:0044550)	55	8.07E-08
	root system development (GO:0022622)	126	9.23E-07
	root development (GO:0048364)	124	1.08E-06
	cell division (GO:0051301)	64	6.71E-06
	cell communication (GO:0007154)	206	8.47E-06
	phenylpropanoid metabolic process (GO:0009698)	25	1.08E-05
	lignin metabolic process (GO:0009808)	17	1.19E-05
	flavonoid biosynthetic process (GO:0009813)	21	1.20E-05
	cell wall modification (GO:0042545)	31	1.23E-05
	carbohydrate catabolic process (GO:0016052)	36	1.26E-05
	auxin transport (GO:0060918)	22	1.77E-05
	plant organ morphogenesis (GO:1905392)	95	1.83E-05
	flavonoid metabolic process (GO:0009812)	29	1.94E-05
	hormone transport (GO:0009914)	22	2.58E-05
	post-embryonic development (GO:0009791)	191	3.34E-05
	phenylpropanoid biosynthetic process (GO:0009699)	20	1.32E-04
	lignin biosynthetic process (GO:0009809)	13	2.43E-04
	phyllome development (GO:0048827)	82	4.55E-04
	shoot system morphogenesis (GO:0010016)	34	4.66E-04
	cell wall organization (GO:0071555)	40	5.72E-04
	reproductive structure development (GO:0048608)	164	5.83E-04
	reproductive system development (GO:0061458)	164	5.87E-04
	root morphogenesis (GO:0010015)	68	9.15E-04
	plant-type cell wall loosening (GO:0009828)	11	1.25E-03
	microtubule-based process (GO:0007017)	31	1.33E-03
	auxin polar transport (GO:0009926)	15	1.37E-03
	phloem or xylem histogenesis (GO:0010087)	25	2.42E-03
	meristem development (GO:0048507)	56	2.75E-03
	anatomical structure arrangement (GO:0048532)	21	3.23E-03
	seed development (GO:0048316)	103	4.11E-03
	pattern specification process (GO:0007389)	37	4.20E-03
	mitotic cell cycle process (GO:1903047)	25	4.35E-03
	mitotic cell cycle (GO:0000278)	30	5.17E-03
	developmental process involved in reproduction (GO:0003006)	173	6.57E-03
	regulation of response to stimulus (GO:0048583)	118	6.61E-03
	microtubule cytoskeleton organization (GO:0000226)	24	6.87E-03
	vesicle-mediated transport (GO:0016192)	12	7.78E-03
	cytokinesis (GO:0000910)	26	7.79E-03
	indole-containing compound metabolic process (GO:0042430)	25	8.39E-03
	salicylic acid catabolic process (GO:0046244)	4	1.27E-02
	polysaccharide metabolic process (GO:0005976)	49	1.41E-02
	monocarboxylic acid catabolic process (GO:0072329)	12	1.47E-02
	plant organ senescence (GO:0090693)	35	2.07E-02
	microtubule-based movement (GO:0007018)	11	2.34E-02
	post-embryonic plant organ development (GO:0090696)	23	2.52E-02
	ethylene-activated signaling pathway (GO:0009873)	11	2.62E-02
	response to gibberellin (GO:0009739)	18	2.64E-02
	meristem initiation (GO:0010014)	10	2.71E-02
	indole-containing compound biosynthetic process (GO:0042435)	17	3.47E-02
	organic anion transport (GO:0015711)	21	3.48E-02

	cellular response to auxin stimulus (GO:0071365)	14	4.41E-02
	homeostatic process (GO:0042592)	54	4.47E-02
	peptidyl-amino acid modification (GO:0018193)	17	4.58E-02
	post-embryonic root development (GO:0048528)	18	4.60E-02
	post-embryonic plant organ morphogenesis (GO:0090697)	17	4.62E-02
	embryonic meristem development (GO:0048508)	8	4.83E-02
L1W vs L0W down	photosynthesis (GO:0015979)	106	5.34E-39
	photosynthesis, light reaction (GO:0019684)	75	1.69E-28
	chloroplast organization (GO:0009658)	71	1.72E-18
	chlorophyll metabolic process (GO:0015994)	54	2.35E-12
	thylakoid membrane organization (GO:0010027)	27	6.39E-11
	photosynthetic electron transport chain (GO:0009767)	25	4.25E-10
	plastid membrane organization (GO:0009668)	27	7.03E-10
	lipid biosynthetic process (GO:0008610)	118	4.04E-09
	photosynthesis, light harvesting (GO:0009765)	21	2.17E-08
	photosynthesis, dark reaction (GO:0019685)	13	3.40E-07
	isoprenoid metabolic process (GO:0006720)	53	2.20E-06
	carbon fixation (GO:0015977)	12	2.25E-06
	terpenoid metabolic process (GO:0006721)	46	2.43E-06
	chlorophyll biosynthetic process (GO:0015995)	23	2.75E-06
	photosynthetic electron transport in photosystem I (GO:0009773)	12	8.65E-06
	starch metabolic process (GO:0005982)	20	1.16E-05
	transmembrane transport (GO:0055085)	111	1.17E-05
	photosystem II assembly (GO:0010207)	14	1.60E-05
	signal transduction (GO:0007165)	221	3.15E-05
	tetraterpenoid metabolic process (GO:0016108)	14	6.58E-05
	carotenoid metabolic process (GO:0016116)	14	6.64E-05
	chloroplast rRNA processing (GO:1901259)	12	7.52E-05
	regulation of photosynthesis, light reaction (GO:0042548)	13	1.25E-04
	signaling (GO:0023052)	222	1.40E-04
	photosystem II repair (GO:0010206)	9	1.97E-04
	photosynthesis, light harvesting in photosystem I (GO:0009768)	11	2.52E-04
	regulation of stomatal closure (GO:0090333)	14	3.33E-04
	carbohydrate biosynthetic process (GO:0016051)	52	3.70E-04
	nonphotochemical quenching (GO:0010196)	9	4.27E-04
	energy quenching (GO:1990066)	9	4.30E-04
	regulation of cellular ketone metabolic process (GO:0010565)	28	6.68E-04
	response to salicylic acid (GO:0009751)	56	6.96E-04
	NADH dehydrogenase complex (plastoquinone) assembly (GO:0010258)	7	7.24E-04
	photomorphogenesis (GO:0009640)	20	7.46E-04
	glucan metabolic process (GO:0044042)	35	8.09E-04
	regulation of post-embryonic development (GO:0048580)	62	8.31E-04
	isoprenoid biosynthetic process (GO:0008299)	33	8.38E-04
	chloroplast RNA processing (GO:0031425)	10	1.38E-03
	photosystem I assembly (GO:0048564)	9	1.51E-03
	plant-type hypersensitive response (GO:0009626)	15	2.02E-03
	programmed cell death induced by symbiont (GO:0034050)	15	2.36E-03
	organic acid transport (GO:0015849)	25	2.73E-03
	protein import into chloroplast thylakoid membrane (GO:0045038)	6	2.75E-03
	regulation of chlorophyll biosynthetic process (GO:0010380)	10	2.93E-03
	biological process involved in interaction with symbiont (GO:0051702)	15	3.16E-03
	regulation of stomatal movement (GO:0010119)	21	3.26E-03
	plastid translation (GO:0032544)	9	3.47E-03
	tetraterpenoid biosynthetic process (GO:0016109)	9	4.28E-03
	carotenoid biosynthetic process (GO:0016117)	9	4.30E-03
	organic acid transmembrane transport (GO:1903825)	18	4.30E-03
	hexose metabolic process (GO:0019318)	18	4.77E-03
	plant organ senescence (GO:0090693)	44	5.08E-03
	regulation of seed germination (GO:0010029)	19	5.75E-03
	leaf development (GO:0048366)	65	5.88E-03

	quinone metabolic process (GO:1901661)	11	5.89E-03
	quinone biosynthetic process (GO:1901663)	11	5.91E-03
	ketone biosynthetic process (GO:0042181)	11	7.04E-03
	hormone-mediated signaling pathway (GO:0009755)	91	8.02E-03
	glucose metabolic process (GO:0006006)	12	8.35E-03
	hexose biosynthetic process (GO:0019319)	9	8.43E-03
	leaf senescence (GO:0010150)	38	8.46E-03
	cellular response to heat (GO:0034605)	16	8.58E-03
	aldonate transmembrane transport (GO:0042873)	5	9.94E-03
	starch biosynthetic process (GO:0019252)	10	1.11E-02
	photosynthetic acclimation (GO:0009643)	6	1.27E-02
	terpenoid biosynthetic process (GO:0016114)	25	1.87E-02
	photosystem II stabilization (GO:0042549)	5	2.22E-02
	calcium ion transport (GO:0006816)	15	2.27E-02
	flavonoid metabolic process (GO:0009812)	24	3.24E-02
	salicylic acid mediated signaling pathway (GO:0009863)	15	3.59E-02
	dephosphorylation (GO:0016311)	27	4.63E-02
L2W vs L0W up	secondary metabolite biosynthetic process (GO:0044550)	74	1.52E-10
	secondary metabolic process (GO:0019748)	121	2.05E-10
	phenylpropanoid metabolic process (GO:0009698)	34	7.56E-08
	lignin metabolic process (GO:0009808)	23	1.28E-07
	lignin biosynthetic process (GO:0009809)	20	2.57E-07
	phenylpropanoid biosynthetic process (GO:0009699)	29	3.31E-07
	amino acid biosynthetic process (GO:0008652)	42	4.05E-07
	cell wall organization or biogenesis (GO:0071554)	130	6.38E-07
	hormone-mediated signaling pathway (GO:0009755)	110	1.10E-05
	root system development (GO:0022622)	150	1.83E-05
	root development (GO:0048364)	148	2.02E-05
	organic anion transport (GO:0015711)	33	1.63E-04
	plant organ morphogenesis (GO:1905392)	112	2.61E-04
	ethylene-activated signaling pathway (GO:0009873)	17	5.10E-04
	carboxylic acid transport (GO:0046942)	27	1.08E-03
	pattern specification process (GO:0007389)	46	3.55E-03
	leaf development (GO:0048366)	69	3.84E-03
	leaf senescence (GO:0010150)	41	3.92E-03
	fluid transport (GO:0042044)	12	4.28E-03
	water transport (GO:0006833)	12	4.32E-03
	anatomical structure morphogenesis (GO:0009653)	173	4.41E-03
	plant-type cell wall organization or biogenesis (GO:0071669)	69	5.83E-03
	cell wall modification (GO:0042545)	29	7.53E-03
	flavonoid biosynthetic process (GO:0009813)	18	8.10E-03
	tissue development (GO:0009888)	196	8.50E-03
	cell wall organization (GO:0071555)	44	9.76E-03
	root morphogenesis (GO:0010015)	79	1.04E-02
	anatomical structure formation involved in morphogenesis (GO:0048646)	40	1.09E-02
	shoot system development (GO:0048367)	157	1.15E-02
	vascular process in circulatory system (GO:0003018)	8	1.78E-02
	vascular transport (GO:0010232)	8	1.79E-02
	phloem transport (GO:0010233)	8	1.80E-02
	hormone transport (GO:0009914)	19	1.80E-02
	auxin transport (GO:0060918)	18	2.67E-02
	flavonoid metabolic process (GO:0009812)	25	2.83E-02
	plant-type secondary cell wall biogenesis (GO:0009834)	17	2.99E-02
	auxin polar transport (GO:0009926)	14	3.49E-02
	lateral root morphogenesis (GO:0010102)	15	3.73E-02
	isopentenyl diphosphate biosynthetic process, mevalonate pathway (GO:0019287)	5	3.74E-02
	jasmonic acid mediated signaling pathway (GO:0009867)	23	3.74E-02
	oligosaccharide biosynthetic process (GO:0009312)	10	4.32E-02
	chorismate metabolic process (GO:0046417)	6	4.55E-02
	inorganic anion transport (GO:0015698)	20	4.55E-02

	tyrosine metabolic process (GO:0006570)	6	4.56E-02
	jasmonic acid metabolic process (GO:0009694)	11	4.60E-02
	coumarin metabolic process (GO:0009804)	5	4.97E-02
	chorismate biosynthetic process (GO:0009423)	5	4.99E-02
L2W vs L0W down	photosynthesis (GO:0015979)	126	6.70E-48
	photosynthesis, light reaction (GO:0019684)	92	6.48E-37
	chloroplast organization (GO:0009658)	78	1.45E-19
	pigment metabolic process (GO:0042440)	94	3.09E-18
	chlorophyll metabolic process (GO:0015994)	65	9.78E-16
	photosynthesis, light harvesting (GO:0009765)	31	2.78E-14
	photosynthetic electron transport chain (GO:0009767)	27	2.17E-10
	regulation of photosynthesis (GO:0010109)	26	2.68E-09
	chlorophyll biosynthetic process (GO:0015995)	30	3.48E-09
	thylakoid membrane organization (GO:0010027)	26	3.57E-09
	photosynthesis, light harvesting in photosystem I (GO:0009768)	18	1.09E-08
	photosystem II assembly (GO:0010207)	18	1.26E-07
	photosynthesis, dark reaction (GO:0019685)	13	1.26E-06
	regulation of photosynthesis, light reaction (GO:0042548)	17	1.27E-06
	photomorphogenesis (GO:0009640)	27	2.39E-06
	photosynthetic electron transport in photosystem I (GO:0009773)	13	4.78E-06
	response to reactive oxygen species (GO:0000302)	38	5.07E-06
	electron transport chain (GO:0022900)	31	5.34E-06
	regulation of stomatal closure (GO:0090333)	18	5.49E-06
	carbon fixation (GO:0015977)	12	6.53E-06
	photosystem II repair (GO:0010206)	11	1.39E-05
	carotenoid biosynthetic process (GO:0016117)	12	1.49E-04
	organonitrogen compound biosynthetic process (GO:1901566)	199	1.56E-04
	nonphotochemical quenching (GO:0010196)	10	1.84E-04
	photosystem I assembly (GO:0048564)	10	7.63E-04
	flavonoid metabolic process (GO:0009812)	30	2.26E-03
	regulation of chlorophyll metabolic process (GO:0090056)	12	3.07E-03
	hormone-mediated signaling pathway (GO:0009755)	102	4.15E-03
	photosynthetic acclimation (GO:0009643)	7	4.67E-03
	regulation of chlorophyll biosynthetic process (GO:0010380)	10	6.21E-03
	photosystem II stabilization (GO:0042549)	6	7.04E-03
	xanthophyll metabolic process (GO:0016122)	8	7.17E-03
	chloroplast localization (GO:0019750)	11	1.11E-02
	vesicle-mediated transport (GO:0016192)	22	1.84E-02
	photoinhibition (GO:0010205)	6	2.01E-02
	negative regulation of photosynthesis, light reaction (GO:0043155)	6	2.02E-02
	shoot system development (GO:0048367)	163	2.24E-02
	PSII associated light-harvesting complex II catabolic process (GO:0010304)	5	3.29E-02
	carotene biosynthetic process (GO:0016120)	5	3.30E-02
	leaf senescence (GO:0010150)	38	4.21E-02
L3W vs L0W up	secondary metabolite biosynthetic process (GO:0044550)	84	1.40E-14
	plant organ development (GO:0099402)	283	1.53E-14
	phenylpropanoid metabolic process (GO:0009698)	40	6.69E-11
	root system development (GO:0022622)	174	1.97E-10
	root development (GO:0048364)	172	2.07E-10
	phenylpropanoid biosynthetic process (GO:0009699)	34	8.15E-10
	lignin metabolic process (GO:0009808)	24	3.25E-08
	lignin biosynthetic process (GO:0009809)	21	6.10E-08
	cell wall organization or biogenesis (GO:0071554)	131	5.23E-07
	plant organ morphogenesis (GO:1905392)	123	2.12E-06
	leaf development (GO:0048366)	81	7.65E-06
	flavonoid biosynthetic process (GO:0009813)	23	7.24E-05
	tissue development (GO:0009888)	213	1.04E-04
	flavonoid metabolic process (GO:0009812)	32	2.30E-04
	lipid metabolic process (GO:0006629)	160	3.09E-04
	auxin transport (GO:0060918)	23	3.42E-04

	auxin polar transport (GO:0009926)	19	3.58E-04
	root morphogenesis (GO:0010015)	87	4.19E-04
	hormone transport (GO:0009914)	23	4.95E-04
	plant-type cell wall organization or biogenesis (GO:0071669)	74	7.35E-04
	cell wall organization (GO:0071555)	48	9.94E-04
	vesicle-mediated transport (GO:0016192)	17	1.76E-03
	regulation of phenylpropanoid metabolic process (GO:2000762)	11	1.96E-03
	shoot system development (GO:0048367)	162	3.55E-03
	L-phenylalanine metabolic process (GO:0006558)	9	3.99E-03
	cell wall modification (GO:0042545)	29	7.59E-03
	plant-type secondary cell wall biogenesis (GO:0009834)	18	9.93E-03
	plant-type cell wall modification (GO:0009827)	15	1.03E-02
	post-embryonic root development (GO:0048528)	24	1.33E-02
	lateral root development (GO:0048527)	23	1.39E-02
	lateral root morphogenesis (GO:0010102)	16	1.81E-02
	pattern specification process (GO:0007389)	43	1.91E-02
	photosynthesis, light reaction (GO:0019684)	2	2.32E-02
	salicylic acid catabolic process (GO:0046244)	4	2.67E-02
	adenine transport (GO:0015853)	4	2.68E-02
	guanine transport (GO:0015854)	4	2.69E-02
	inorganic anion transport (GO:0015698)	21	2.75E-02
	plant-type cell wall organization (GO:0009664)	28	3.32E-02
	phloem or xylem histogenesis (GO:0010087)	26	3.37E-02
	photosynthesis (GO:0015979)	6	3.49E-02
	shoot system morphogenesis (GO:0010016)	34	3.80E-02
L3W vs L0W down	photosynthesis (GO:0015979)	136	5.09E-50
	photosynthesis, light reaction (GO:0019684)	97	4.54E-37
	chlorophyll metabolic process (GO:0015994)	72	2.57E-17
	photosynthesis, light harvesting (GO:0009765)	32	5.67E-14
	thylakoid membrane organization (GO:0010027)	30	8.39E-11
	chlorophyll biosynthetic process (GO:0015995)	34	1.48E-10
	photosynthetic electron transport chain (GO:0009767)	28	3.63E-10
	regulation of photosynthesis (GO:0010109)	27	4.04E-09
	photosystem II assembly (GO:0010207)	20	2.38E-08
	photosynthesis, light harvesting in photosystem I (GO:0009768)	18	4.18E-08
	lipid biosynthetic process (GO:0008610)	132	2.21E-07
	regulation of photosynthesis, light reaction (GO:0042548)	18	1.02E-06
	photosynthesis, dark reaction (GO:0019685)	13	3.41E-06
	photosystem II repair (GO:0010206)	12	6.60E-06
	carotenoid metabolic process (GO:0016116)	17	1.09E-05
	carbon fixation (GO:0015977)	12	1.70E-05
	regulation of stomatal closure (GO:0090333)	18	2.04E-05
	photosynthetic electron transport in photosystem I (GO:0009773)	12	6.13E-05
	photomorphogenesis (GO:0009640)	24	2.68E-04
	carotenoid biosynthetic process (GO:0016117)	12	3.56E-04
	chloroplast RNA processing (GO:0031425)	12	4.74E-04
	regulation of chlorophyll biosynthetic process (GO:0010380)	12	1.11E-03
	photosystem I assembly (GO:0048564)	10	1.62E-03
	nonphotochemical quenching (GO:0010196)	9	1.77E-03
	energy quenching (GO:1990066)	9	1.78E-03
	photosynthetic acclimation (GO:0009643)	7	7.76E-03
	PSII associated light-harvesting complex II catabolic process (GO:0010304)	6	1.10E-02
	cytochrome b6f complex assembly (GO:0010190)	6	1.10E-02
	photosystem II stabilization (GO:0042549)	6	1.11E-02
	system development (GO:0048731)	411	1.45E-02
	photoprotection (GO:0010117)	7	1.97E-02
	photosynthetic electron transport in photosystem II (GO:0009772)	6	2.21E-02
	photoinhibition (GO:0010205)	6	2.96E-02
	negative regulation of photosynthesis, light reaction (GO:0043155)	6	2.97E-02

0W, 1W, 2W and 3W indicate weeks after plantation.

Table S3. Gene ontology (GO) analysis of up- and down-regulated differentially expressed genes (DEGs) in high rooting group (H) compared to low group 0 week after plantation (H0W).

	**GO biological process complete**	**Gene**	**FDR**
H1W vs H0W up	tissue development (GO:0009888)	188	2.54E-07
	cell wall organization or biogenesis (GO:0071554)	110	3.10E-07
	plant organ development (GO:0099402)	198	1.34E-06
	system development (GO:0048731)	311	1.44E-05
	plant organ morphogenesis (GO:1905392)	95	1.01E-04
	shoot system development (GO:0048367)	138	4.08E-04
	growth (GO:0040007)	125	5.02E-04
	root system development (GO:0022622)	116	6.11E-04
	root development (GO:0048364)	114	8.61E-04
	cell wall modification (GO:0042545)	27	1.57E-03
	amino acid transport (GO:0006865)	15	1.72E-03
	signal transduction (GO:0007165)	178	1.77E-03
	root morphogenesis (GO:0010015)	69	2.20E-03
	secondary metabolic process (GO:0019748)	76	2.44E-03
	anatomical structure formation involved in morphogenesis (GO:0048646)	36	2.86E-03
	secondary metabolite biosynthetic process (GO:0044550)	43	2.93E-03
	pattern specification process (GO:0007389)	39	2.96E-03
	phloem or xylem histogenesis (GO:0010087)	25	4.20E-03
	radial pattern formation (GO:0009956)	10	5.46E-03
	circulatory system process (GO:0003013)	8	6.14E-03
	vascular process in circulatory system (GO:0003018)	8	6.18E-03
	vascular transport (GO:0010232)	8	6.22E-03
	carboxylic acid transport (GO:0046942)	21	6.22E-03
	phloem transport (GO:0010233)	8	6.26E-03
	sterol metabolic process (GO:0016125)	14	6.27E-03
	syncytium formation (GO:0006949)	7	6.32E-03
	phenylpropanoid metabolic process (GO:0009698)	19	8.08E-03
	leaf development (GO:0048366)	56	8.09E-03
	plant-type cell wall organization or biogenesis (GO:0071669)	57	8.87E-03
	fluid transport (GO:0042044)	10	9.49E-03
	water transport (GO:0006833)	10	9.55E-03
	lignin metabolic process (GO:0009808)	12	1.00E-02
	intracellular transport (GO:0046907)	20	1.03E-02
	organic acid transport (GO:0015849)	21	1.05E-02
	lipid modification (GO:0030258)	15	1.06E-02
	plant-type cell wall modification (GO:0009827)	13	1.13E-02
	shoot system morphogenesis (GO:0010016)	30	1.46E-02
	lipid oxidation (GO:0034440)	11	1.54E-02
	lipid catabolic process (GO:0016042)	18	1.57E-02
	organic anion transport (GO:0015711)	23	1.79E-02
	cell division (GO:0051301)	52	1.99E-02
	regulation of phenylpropanoid metabolic process (GO:2000762)	8	2.17E-02
	plant-type cell wall loosening (GO:0009828)	9	2.22E-02
	brassinosteroid mediated signaling pathway (GO:0009742)	12	2.96E-02
	phenylpropanoid biosynthetic process (GO:0009699)	15	2.98E-02
	primary meristem tissue development (GO:0010065)	5	3.38E-02
	lignin biosynthetic process (GO:0009809)	9	4.68E-02
	aromatic amino acid metabolic process (GO:0009072)	20	4.80E-02
	cell wall organization (GO:0071555)	34	4.95E-02
H1W vs H0W down	photosynthesis, light reaction (GO:0019684)	92	3.40E-41
	chloroplast organization (GO:0009658)	84	2.24E-26
	chlorophyll metabolic process (GO:0015994)	63	2.93E-17
	lipid metabolic process (GO:0006629)	195	6.93E-14
	photosynthetic electron transport chain (GO:0009767)	30	1.12E-13
	photosynthesis, light harvesting (GO:0009765)	28	2.18E-13
	pigment biosynthetic process (GO:0046148)	52	6.24E-13
	regulation of photosynthesis (GO:0010109)	28	7.65E-12

	thylakoid membrane organization (GO:0010027)	27	5.51E-11
	chlorophyll biosynthetic process (GO:0015995)	30	1.95E-10
	lipid biosynthetic process (GO:0008610)	120	6.63E-10
	photosystem II assembly (GO:0010207)	19	3.78E-09
	terpenoid metabolic process (GO:0006721)	51	1.95E-08
	electron transport chain (GO:0022900)	33	4.44E-08
	isoprenoid metabolic process (GO:0006720)	57	4.80E-08
	photosynthesis, light harvesting in photosystem I (GO:0009768)	16	5.55E-08
	chloroplast RNA processing (GO:0031425)	16	8.49E-08
	photosynthetic electron transport in photosystem I (GO:0009773)	14	2.13E-07
	regulation of photosynthesis, light reaction (GO:0042548)	17	2.19E-07
	photosynthesis, dark reaction (GO:0019685)	13	3.05E-07
	carbon fixation (GO:0015977)	12	2.00E-06
	photomorphogenesis (GO:0009640)	24	8.53E-06
	photosystem II repair (GO:0010206)	10	3.14E-05
	cell cycle (GO:0007049)	19	3.16E-05
	NAD(P)H dehydrogenase complex assembly (GO:0010275)	9	7.44E-05
	tissue development (GO:0009888)	94	1.19E-03
	photosystem I assembly (GO:0048564)	9	1.39E-03
	flavonoid metabolic process (GO:0009812)	28	1.44E-03
	nonphotochemical quenching (GO:0010196)	8	1.89E-03
	energy quenching (GO:1990066)	8	1.90E-03
	immune system process (GO:0002376)	38	2.12E-03
	macromolecule metabolic process (GO:0043170)	673	2.19E-03
	photosynthetic acclimation (GO:0009643)	7	2.36E-03
	mitotic cell cycle (GO:0000278)	3	3.45E-03
	cytochrome b6f complex assembly (GO:0010190)	6	3.77E-03
	NADH dehydrogenase complex (plastoquinone) assembly (GO:0010258)	6	3.79E-03
	photosystem II stabilization (GO:0042549)	6	3.81E-03
	meiotic cell cycle (GO:0051321)	6	5.56E-03
	embryo development (GO:0009790)	84	6.49E-03
	meiotic cell cycle process (GO:1903046)	5	6.65E-03
	photoinhibition (GO:0010205)	6	1.13E-02
	negative regulation of photosynthesis, light reaction (GO:0043155)	6	1.14E-02
	embryo development ending in seed dormancy (GO:0009793)	77	1.36E-02
	carotenoid biosynthetic process (GO:0016117)	8	1.42E-02
	mitotic cell cycle process (GO:1903047)	2	1.73E-02
	seed development (GO:0048316)	118	1.74E-02
	reproductive structure development (GO:0048608)	184	1.74E-02
	PSII associated light-harvesting complex II catabolic process (GO:0010304)	5	1.97E-02
	fruit development (GO:0010154)	121	2.43E-02
	ribulose bisphosphate carboxylase complex assembly (GO:0110102)	5	2.67E-02
	phenylpropanoid biosynthetic process (GO:0009699)	17	2.81E-02
	lignin biosynthetic process (GO:0009809)	10	3.51E-02
	photosynthetic electron transport in photosystem II (GO:0009772)	5	3.61E-02
	phenylpropanoid metabolic process (GO:0009698)	19	4.51E-02
H2W vs H0W up	tissue development (GO:0009888)	390	7.88E-21
	cell division (GO:0051301)	147	3.00E-17
	plant organ development (GO:0099402)	396	3.57E-16
	cell cycle process (GO:0022402)	145	2.30E-12
	root development (GO:0048364)	237	5.16E-11
	root system development (GO:0022622)	240	5.40E-11
	cell wall organization or biogenesis (GO:0071554)	196	1.90E-10
	plant organ morphogenesis (GO:1905392)	182	3.74E-09
	pattern specification process (GO:0007389)	84	1.08E-08
	mitotic cell cycle process (GO:1903047)	56	1.23E-07
	mitotic cell cycle (GO:0000278)	65	3.04E-07
	plant-type cell wall organization or biogenesis (GO:0071669)	116	7.89E-07
	meristem development (GO:0048507)	112	2.37E-06
	root morphogenesis (GO:0010015)	128	1.00E-05

	shoot system development (GO:0048367)	247	1.75E-05
	phenylpropanoid metabolic process (GO:0009698)	37	2.94E-05
	phloem or xylem histogenesis (GO:0010087)	46	5.29E-05
	macromolecule metabolic process (GO:0043170)	1061	8.59E-05
	phenylpropanoid biosynthetic process (GO:0009699)	31	8.65E-05
	plant-type secondary cell wall biogenesis (GO:0009834)	29	1.46E-04
	shoot system morphogenesis (GO:0010016)	56	2.24E-04
	secondary metabolite biosynthetic process (GO:0044550)	74	2.77E-04
	macromolecule biosynthetic process (GO:0009059)	328	3.01E-04
	lignin metabolic process (GO:0009808)	21	5.25E-04
	plant-type cell wall biogenesis (GO:0009832)	60	6.81E-04
	cell wall biogenesis (GO:0042546)	83	6.98E-04
	cell differentiation (GO:0030154)	203	7.08E-04
	signaling (GO:0023052)	319	1.42E-03
	lignin biosynthetic process (GO:0009809)	17	1.70E-03
	hormone transport (GO:0009914)	28	2.56E-03
	auxin transport (GO:0060918)	27	3.71E-03
	radial pattern formation (GO:0009956)	14	6.10E-03
	meiotic cell cycle process (GO:1903046)	55	7.15E-03
	carbohydrate biosynthetic process (GO:0016051)	67	7.49E-03
	mitotic nuclear division (GO:0140014)	16	7.62E-03
	flavonoid metabolic process (GO:0009812)	36	8.73E-03
	meiotic cell cycle (GO:0051321)	59	8.76E-03
	spindle organization (GO:0007051)	20	9.70E-03
	photosynthesis (GO:0015979)	10	1.30E-02
	auxin polar transport (GO:0009926)	20	1.31E-02
	flavonoid biosynthetic process (GO:0009813)	23	1.41E-02
	flower development (GO:0009908)	89	1.41E-02
	xylem development (GO:0010089)	17	1.56E-02
	xylan metabolic process (GO:0045491)	19	1.69E-02
	cell wall organization (GO:0071555)	58	1.74E-02
	nuclear division (GO:0000280)	32	2.07E-02
	microtubule cytoskeleton organization involved in mitosis (GO:1902850)	12	2.10E-02
	cell wall macromolecule catabolic process (GO:0016998)	12	2.54E-02
	organic anion transport (GO:0015711)	35	2.91E-02
	water transport (GO:0006833)	13	2.92E-02
	root cap development (GO:0048829)	8	3.33E-02
	reproductive structure development (GO:0048608)	274	3.93E-02
	cell wall polysaccharide catabolic process (GO:0044347)	8	4.17E-02
	reproductive system development (GO:0061458)	274	4.29E-02
	xylem and phloem pattern formation (GO:0010051)	22	4.31E-02
	regulation of organelle assembly (GO:1902115)	7	4.38E-02
H2W vs H0W down	photosystem II repair (GO:0010206)	12	4.88E-05
	NAD(P)H dehydrogenase complex assembly (GO:0010275)	10	3.55E-04
	chloroplast mRNA processing (GO:0010239)	9	9.61E-04
	photosystem II stabilization (GO:0042549)	7	6.39E-03
	PSII associated light-harvesting complex II catabolic process (GO:0010304)	7	6.37E-03
	NADH dehydrogenase complex (plastoquinone) assembly (GO:0010258)	7	6.34E-03
	cytochrome b6f complex assembly (GO:0010190)	7	6.32E-03
	carbon fixation (GO:0015977)	13	3.02E-05
	photosynthesis, dark reaction (GO:0019685)	13	2.99E-05
	photosystem II assembly (GO:0010207)	25	5.44E-10
	reductive pentose-phosphate cycle (GO:0019253)	12	7.81E-05
	protein import into chloroplast thylakoid membrane (GO:0045038)	6	1.58E-02
	photosynthesis, light harvesting in photosystem I (GO:0009768)	19	2.23E-07
	energy quenching (GO:1990066)	12	1.21E-04
	photosynthetic electron transport in photosystem I (GO:0009773)	14	2.82E-05
	cellular response to light intensity (GO:0071484)	10	8.84E-04
	photosynthesis, light harvesting (GO:0009765)	33	2.76E-12
	photosynthetic acclimation (GO:0009643)	8	5.79E-03

	photoprotection (GO:0010117)	10	1.32E-03
	regulation of photosynthesis, light reaction (GO:0042548)	22	9.94E-08
	chloroplast RNA processing (GO:0031425)	17	5.36E-06
	photosynthetic electron transport chain (GO:0009767)	35	3.18E-12
	photosynthesis, light reaction (GO:0019684)	113	1.81E-39
	circadian regulation of gene expression (GO:0032922)	8	8.03E-03
	photosynthesis (GO:0015979)	156	1.24E-51
	thylakoid membrane organization (GO:0010027)	34	6.11E-11
	chloroplast rRNA processing (GO:1901259)	15	7.27E-05
	regulation of photosynthesis (GO:0010109)	33	1.61E-10
	photosystem I assembly (GO:0048564)	11	2.09E-03
	carotenoid biosynthetic process (GO:0016117)	13	6.49E-04
	tetraterpenoid biosynthetic process (GO:0016109)	13	6.46E-04
	response to high light intensity (GO:0009644)	37	8.07E-11
	carotenoid metabolic process (GO:0016116)	19	1.38E-05
	chlorophyll biosynthetic process (GO:0015995)	41	6.90E-12
	response to photooxidative stress (GO:0080183)	7	3.55E-02
	protoporphyrinogen IX biosynthetic process (GO:0006782)	8	1.96E-02
	protoporphyrinogen IX metabolic process (GO:0046501)	8	1.96E-02
	xanthophyll metabolic process (GO:0016122)	9	1.12E-02
	regulation of chlorophyll biosynthetic process (GO:0010380)	14	6.48E-04
	phosphate ion transmembrane transport (GO:0035435)	7	4.56E-02
	negative regulation of photosynthesis (GO:1905156)	7	4.55E-02
	chloroplast organization (GO:0009658)	112	3.02E-28
	oxylipin biosynthetic process (GO:0031408)	10	1.72E-02
	regulation of chlorophyll metabolic process (GO:0090056)	15	1.71E-03
	response to low light intensity stimulus (GO:0009645)	14	3.03E-03
	chloroplast fission (GO:0010020)	10	2.11E-02
	chlorophyll metabolic process (GO:0015994)	85	1.30E-18
	oxylipin metabolic process (GO:0031407)	10	2.61E-02
	quinone biosynthetic process (GO:1901663)	14	6.60E-03
	quinone metabolic process (GO:1901661)	14	6.57E-03
	cellular response to light stimulus (GO:0071482)	69	2.28E-13
	pigment biosynthetic process (GO:0046148)	69	2.77E-13
	lignin biosynthetic process (GO:0009809)	16	3.41E-03
	NADPH regeneration (GO:0006740)	13	1.12E-02
	electron transport chain (GO:0022900)	43	7.07E-08
	translational elongation (GO:0006414)	14	9.39E-03
	plastid localization (GO:0051644)	13	1.59E-02
	regulation of circadian rhythm (GO:0042752)	23	3.85E-04
	NADP metabolic process (GO:0006739)	16	5.80E-03
	response to far red light (GO:0010218)	19	2.01E-03
	response to light intensity (GO:0009642)	133	4.85E-23
	regulation of stomatal closure (GO:0090333)	14	1.33E-02
	pigment metabolic process (GO:0042440)	118	3.38E-19
	circadian rhythm (GO:0007623)	42	2.74E-06
	cellular response to red or far red light (GO:0071489)	18	8.38E-03
	lignin metabolic process (GO:0009808)	17	1.16E-02
	phenylpropanoid biosynthetic process (GO:0009699)	25	6.97E-03
	embryo development (GO:0009790)	116	4.09E-02
H3W vs H0W up	isopentenyl diphosphate biosynthetic process, mevalonate pathway (GO:0019287)	6	3.99E-02
	phloem transport (GO:0010233)	9	4.30E-02
	vascular transport (GO:0010232)	9	4.28E-02
	oxylipin biosynthetic process (GO:0031408)	10	1.94E-02
	oxylipin metabolic process (GO:0031407)	10	2.94E-02
	water transport (GO:0006833)	14	1.02E-02
	fluid transport (GO:0042044)	14	1.01E-02
	potassium ion transport (GO:0006813)	14	4.82E-02
	spindle assembly (GO:0051225)	15	2.24E-02
	phenol-containing compound metabolic process (GO:0018958)	17	2.72E-03

	xylem development (GO:0010089)	17	1.26E-02
	spindle organization (GO:0007051)	19	1.46E-02
	auxin polar transport (GO:0009926)	19	2.07E-02
	lignin biosynthetic process (GO:0009809)	21	1.64E-05
	microtubule-based movement (GO:0007018)	22	1.95E-04
	flavonoid biosynthetic process (GO:0009813)	23	1.23E-02
	auxin transport (GO:0060918)	27	1.85E-03
	hormone transport (GO:0009914)	28	1.29E-03
	phenylpropanoid biosynthetic process (GO:0009699)	35	1.25E-06
	flavonoid metabolic process (GO:0009812)	36	6.73E-03
	phenylpropanoid metabolic process (GO:0009698)	42	1.60E-07
	mitotic cell cycle process (GO:1903047)	42	1.27E-03
	phloem or xylem histogenesis (GO:0010087)	43	1.73E-04
	mitotic cell cycle (GO:0000278)	49	3.66E-03
	shoot system morphogenesis (GO:0010016)	49	6.44E-03
	cell wall organization (GO:0071555)	59	8.27E-03
	plant organ senescence (GO:0090693)	68	1.33E-04
	secondary metabolite biosynthetic process (GO:0044550)	85	2.12E-07
	cell wall biogenesis (GO:0042546)	86	7.31E-05
	reproductive shoot system development (GO:0090567)	100	6.66E-04
	cell division (GO:0051301)	117	7.09E-09
	plant-type cell wall organization or biogenesis (GO:0071669)	122	7.25E-09
	root morphogenesis (GO:0010015)	134	1.36E-07
	hormone-mediated signaling pathway (GO:0009755)	157	3.23E-07
	cell wall organization or biogenesis (GO:0071554)	195	7.01E-11
	root development (GO:0048364)	242	6.91E-13
	root system development (GO:0022622)	245	5.29E-13
	reproductive structure development (GO:0048608)	269	4.40E-02
H3W vs H0W down	photosynthesis, light reaction (GO:0019684)	114	6.52E-41
	photosynthesis (GO:0015979)	155	4.11E-52
	response to light intensity (GO:0009642)	129	4.38E-22
	chlorophyll metabolic process (GO:0015994)	82	1.03E-17
	pigment biosynthetic process (GO:0046148)	69	1.04E-13
	photosynthetic electron transport chain (GO:0009767)	37	1.06E-13
	photosynthesis, light harvesting (GO:0009765)	32	7.18E-12
	cellular response to light stimulus (GO:0071482)	63	3.51E-11
	chlorophyll biosynthetic process (GO:0015995)	39	4.72E-11
	photosystem II assembly (GO:0010207)	25	3.58E-10
	thylakoid membrane organization (GO:0010027)	32	5.21E-10
	electron transport chain (GO:0022900)	47	6.42E-10
	regulation of photosynthesis, light reaction (GO:0042548)	22	6.87E-08
	response to blue light (GO:0009637)	54	8.57E-08
	photosynthesis, light harvesting in photosystem I (GO:0009768)	19	1.55E-07
	photosynthetic electron transport in photosystem I (GO:0009773)	15	5.48E-06
	protein targeting to chloroplast (GO:0045036)	24	6.46E-06
	chloroplast RNA processing (GO:0031425)	16	1.56E-05
	rhythmic process (GO:0048511)	39	1.56E-05
	photosynthesis, dark reaction (GO:0019685)	13	2.34E-05
	photomorphogenesis (GO:0009640)	30	2.34E-05
	carbon fixation (GO:0015977)	13	2.35E-05
	carotenoid metabolic process (GO:0016116)	18	3.37E-05
	photosystem II repair (GO:0010206)	11	1.75E-04
	NAD(P)H dehydrogenase complex assembly (GO:0010275)	10	2.93E-04
	photosystem I assembly (GO:0048564)	12	4.62E-04
	regulation of chlorophyll biosynthetic process (GO:0010380)	14	4.85E-04
	photosynthetic electron transport in photosystem II (GO:0009772)	9	7.48E-04
	carbohydrate transport (GO:0008643)	21	1.12E-03
	regulation of chlorophyll metabolic process (GO:0090056)	15	1.20E-03
	photosynthetic acclimation (GO:0009643)	8	4.71E-03
	NADH dehydrogenase complex (plastoquinone) assembly (GO:0010258)	7	5.37E-03

photosystem II stabilization (GO:0042549)	7	5.39E-03
lignin biosynthetic process (GO:0009809)	15	6.39E-03
lignin metabolic process (GO:0009808)	17	9.39E-03
xanthophyll metabolic process (GO:0016122)	9	9.54E-03
mitotic cell cycle (GO:0000278)	9	9.81E-03
negative regulation of photosynthesis, light reaction (GO:0043155)	7	1.68E-02
photoinhibition (GO:0010205)	7	1.68E-02
secondary metabolite biosynthetic process (GO:0044550)	61	3.41E-02

0W, 1W, 2W and 3W indicate weeks after plantation.

Table S4. Comparison of gene expression between high- and low-rooting groups based on RNA-seq and qRT-PCR analyses.

	RNA-seq (High)	RNA-seq (Low)	RNA-seq log_2FC	qRT-PCR (High)	qRT-PCR (Low)	qRT-PCR log_2FC
TRINITY_DN5565_c0_g1_i3	40.6±29.8.	0.0±0.0	10.8	9.1±0.9	13.5±2.3	4.40
TRINITY_DN10757_c0_g1_i1	119.9±15.5	36.6±5.8	1.66	6.3±2.6	8.8±0.2	2.55
TRINITY_DN16489_c0_g1_i2	6.8±3.0	1.2±0.4	-2.52	12.9±0.7	10.5±1.2	-2.43
TRINITY_DN3440_c0_g1_i15	0.0±0.0	3.2±0.7	-8.28	9.3±0.6	7.6±0.6	-1.68
TRINITY_DN6189_c0_g1_i11	1.3±0.6	8.1±4.1	-2.69	10.6±0.8	8.8±0.3	-1.86
TRINITY_DN4210_c0_g1_i63	14.3±9.3	0.7±0.2	4.19	6.5±0.4	8.8±3.2	2.33
TRINITY_DN10348_c0_g1_i1	87.1±9.0	29.1±6.7	1.54	6.5±4.6	5.0±0.2	-1.52

FPKM values represent normalized transcript abundance from RNA-seq. qRT-PCR log_2 fold changes were calculated using the –ΔΔCt method, and results are presented as mean ± standard deviation (SD) from three biological replicates.

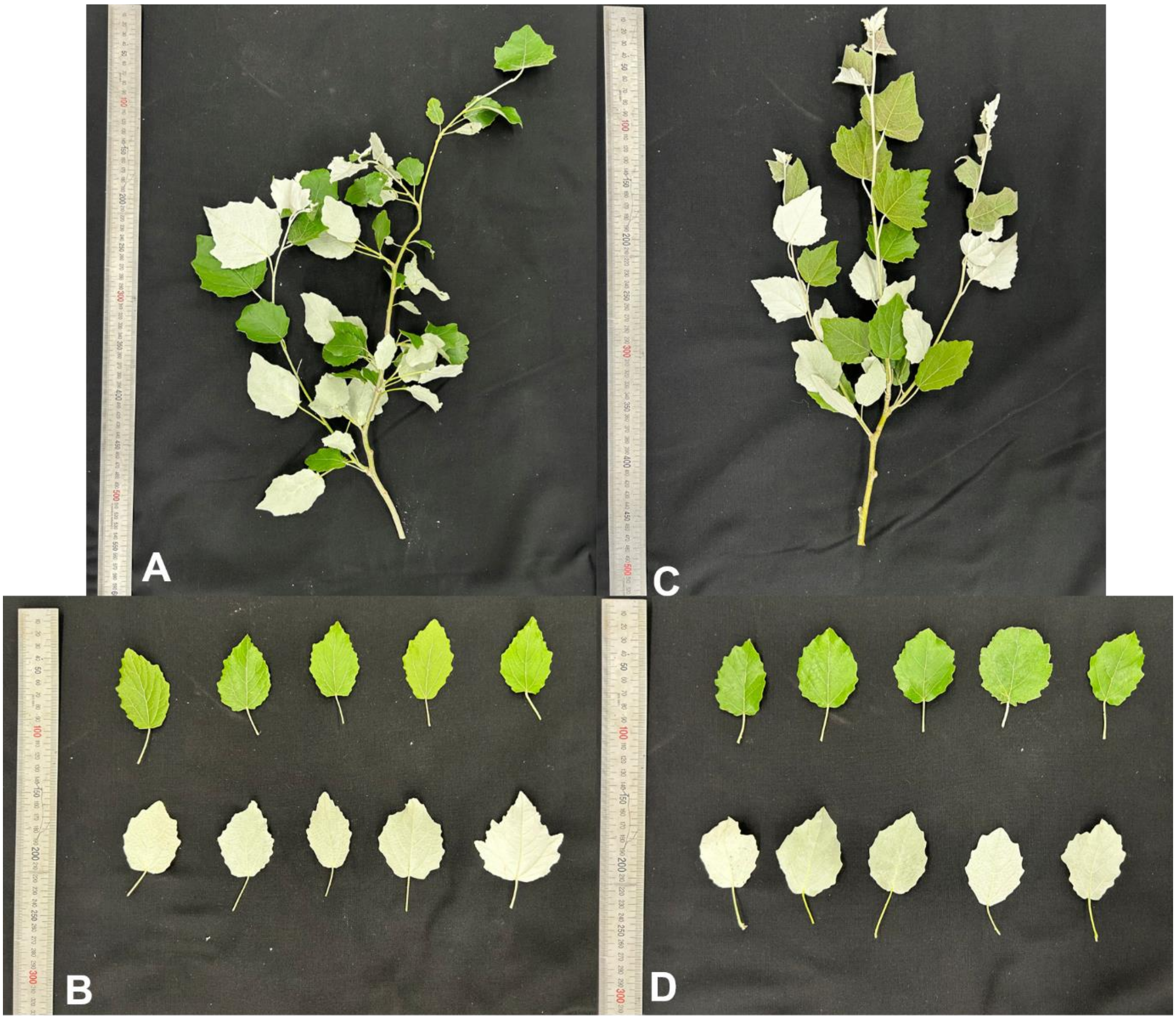

Figure S1. Representative images of (A) branch and leaves of high rooting group, (B) leaf of the high rooting group, (C) branch and leaves of the low rooting group, and (D) leaf of the low rooting group.
